# Reviewing the Evidence Base for the Children and Young People Safety Thermometer (CYPST): A Mixed Studies Review

**DOI:** 10.3390/healthcare4010008

**Published:** 2016-01-11

**Authors:** Lydia Aston, Caron Eyre, Michelle McLoughlin, Rachel Shaw

**Affiliations:** 1 School of Life & Health Sciences, Aston University, Birmingham B4 7ET, UK; astonlr@aston.ac.uk; 2 Birmingham Children’s Hospital Foundation NHS Trust, Steelhouse Lane, Birmingham B4 6NH, UK; caron.eyre@bch.nhs.uk (C.E.); michelle.mcloughlin@bch.nhs.uk (M.M.)

**Keywords:** pediatrics, patient safety, safety thermometer, risk assessment, mixed studies review

## Abstract

The objective was to identify evidence to support use of specific harms for the development of a children and young people’s safety thermometer (CYPST). We searched PubMed, Web of Knowledge, and Cochrane Library post-1999 for studies in pediatric settings about pain, skin integrity, extravasation injury, and use of pediatric early warning scores (PEWS). Following screening, nine relevant articles were included. Convergent synthesis methods were used drawing on thematic analysis to combine findings from studies using a range of methods (qualitative, quantitative, and mixed methods). A review of PEWS was identified so other studies on this issue were excluded. No relevant studies about extravasation injury were identified. The synthesized results therefore focused on pain and skin integrity. Measurement and perception of pain were complex and not always carried out according to best practice. Skin abrasions were common and mostly associated with device related injuries. The findings demonstrate a need for further work on perceptions of pain and effective communication of concerns about pain between parents and nursing staff. Strategies for reducing device-related injuries warrant further research focusing on prevention. Together with the review of PEWS, these synthesized findings support the inclusion of pain, skin integrity, and PEWS in the CYPST.

## 1. Introduction

In 2012 the English National Health Service (NHS) included the Safety Thermometer (ST) [1,2] in the incentivizing scheme, Commissioning for Quality and Innovation (CQUIN), in all NHS healthcare providers, to establish a monthly audit of four common harms: pressure ulcers, falls, catheter associated urinary tract infections, and venous thromboembolism [3]. Auditing harms which apply broadly across healthcare settings initially aimed to detect harms early to prevent further complications and later to reduce the incidence of such harms [4]. The ST offers a quick, standardized method of monitoring and recording patient safety [1,5]. The ST has demonstrated its value as a “harm free” point of care measurement tool in adult settings [4] but there is no equivalent tool in pediatric settings for neonates, children, and young people. This paper reports a review of evidence exploring potential harms to be included in a pediatric version of the ST.

A group of specialist clinicians lead by Birmingham Children’s Hospital NHS Foundation Trust (BCHNHSFT) was formed to determine which harms would be appropriate for pediatric settings. Based on clinical expert consensus and regular audits of hospital databases a group of four harms was identified: Pain with focus on the failure to complete and/or act on pain scores; Skin Integrity, in terms of where there has been failure to complete and/or act on a reliable skin integrity assessment including pressure ulcers and moisture lesions; Extravasation harms, which is related to the failure to observe extravasation for those patients that have a cannula in-situ; and failure to escalate the care of a deteriorating child via the Pediatric Early Warning Scores (PEWS) system [6,7,8,9,10]. This review was conducted to establish whether there was evidence to support the inclusion of the four groups of harms identified by the expert working party and to provide feedback on their appropriateness for inclusion in a pediatric ST.

This review was commissioned by Birmingham Children’s Hospital to examine the evidence base for the selection of harms as the basis of a Children and Young People’s Safety Thermometer (CYPST). The review questions were: is there evidence to support the selection of four harms identified; are these harms currently monitored in practice, and if so how; what are clinicians’ experiences of monitoring these harms?

## 2. Method

A mixed studies review methodology was adopted [11] because the review question was not suited to a systematic review or meta-analysis and its aim was to explore a range of evidence using different methods related to the appropriateness and feasibility of monitoring these harms in practice. Mixed studies reviews enable the exploration of contextual and behavioral factors in the development and piloting of complex tools like a pediatric safety thermometer [11].

### 2.1. Data Sources

PubMed, Web of Knowledge, and Cochrane Library were searched in February 2014 with restrictions to English and post-1999. Searches were updated in February 2015.

### 2.2. Search Strategy

Keywords and phrases for each harm were paired with search terms relating to context and methods. The full search strategy is included in Table 1.

**Table 1:** Search strategy.

Topic	Search Terms
Pain	“pain score* OR measurement” OR pain OR “acute pain” OR “visceral pain”
Skin integrity	skin NEAR/3 integrity OR “pressure ulcer”
Extravasation	Extravasation * OR extravas * OR cannula
PEWS	PEWS OR neonatal OR p$ediatric early warning score* OR “rapid response system” OR “track and trigger aggregate score” OR “early warning score” OR “early warning system” OR “heart rate” OR “blood pressure” OR “blood gas result*”

### 2.3. Inclusion Criteria

Studies included were set in pediatric hospitals or departments; staff/parental measurements or evaluations of pain, skin integrity, extravasation injuries, and/or PEWS; used any method including randomized controlled trials, intervention studies, and studies using quantitative and/or qualitative data; since 1999.

### 2.4. Critical Appraisal

Studies were appraised using the Mixed Methods Appraisal Tool (MMAT), specifically designed to appraise studies using a range of methods [11].

### 2.5. Data Extraction and Synthesis

Data were extracted in the following ways: for qualitative papers, reported theme or categories including data extracts and author commentary were copied verbatim into a spreadsheet; for quantitative and mixed methods papers, descriptive summaries of numerical data presented in tables or figures were written and included in the spreadsheet alongside author commentary reported. A convergent synthesis approach [12] was adopted using qualitative thematic analysis [13,14]. This involved the construction of a matrix of all reported themes and descriptive summaries from all papers and systematic cross-comparisons between them. Themes were then coded, commonalities explored, and new themes generated to represent the findings across the whole set of papers. Initial analyses were conducted by Lydia Aston; emergent themes were examined independently by Lydia Aston and Rachel Shaw who then agreed on the final set.

## 3. Results and Discussion

### 3.1. Study Selection

**Figure 1:**
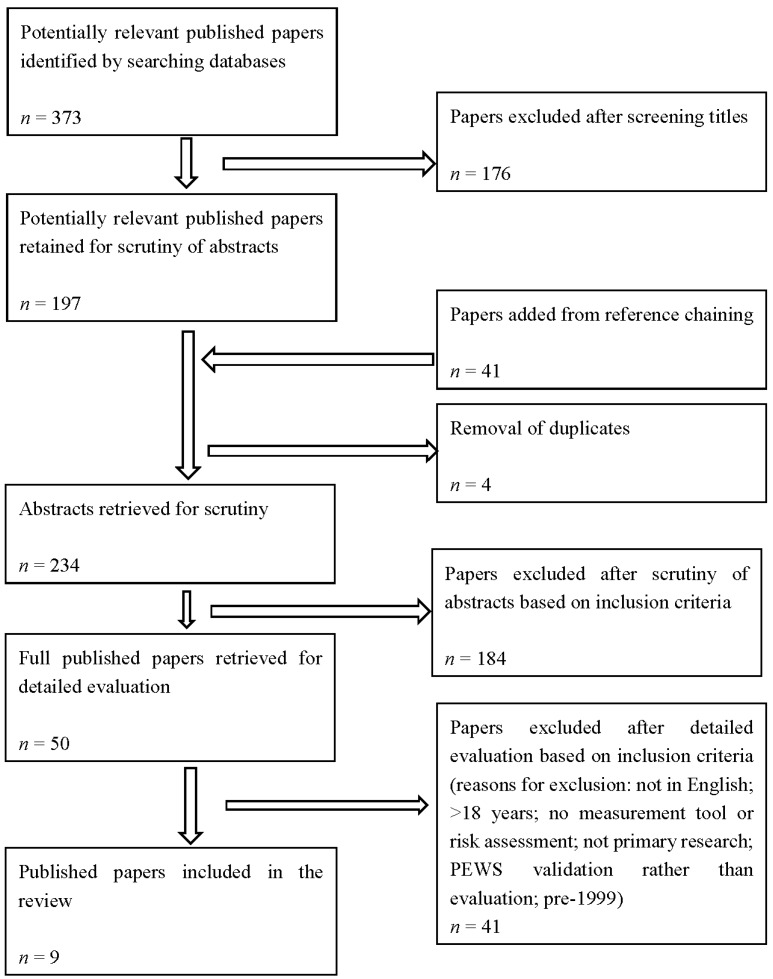
PRISMA flow diagram of screening process.

The search yielded 373 references, which were screened according to the inclusion criteria (see Figure 1). A second researcher reviewed a quarter of records to ensure consistency. If uncertain, discussions were held involving the team member with clinical expertise to determine whether to include or exclude. Reference chaining of included studies was conducted. Papers were screened by title and then abstract. In the final set of papers there were three about pain, six about skin integrity, none about extravasation exclusively, and none about PEWS. Studies about extravasation focused on comparisons of drugs administered by canula rather than harms resulting from them *in situ*. This version of the CYPST was not developed to focus on harms related to the administration of drugs specifically; that is recognized as a significant area of practice requiring its own safety audit tool, especially given that many drugs for adults are not licensed for pediatric use. Some skin integrity papers did cover extravasation, however, because some pressure ulcers were related to intravenous catheters. The studies about PEWS focused on the validation of the PEWS tools rather than their evaluation or nurses’ experiences of using them. Furthermore, an existing systematic review [15] of PEWS tools was identified which already synthesizes the literature specific to the use of PEWS. The findings from the systematic review will be considered alongside the synthesized findings of included studies in the discussion. Searches were re-run in 2015 but no further studies were identified. Nine studies were included in the review (see Table 2).

### 3.2. Study Quality

The quality of included studies varied (a summary quality rating is in Table 2; see the supplementary file for full appraisal details). Transparency of recruitment procedures [16] and the appropriateness of measurements/instruments used was lacking [17]. Some papers failed to include response and follow-up rates [16,17,18,19]. Papers judged higher quality [20,21,22,23] were given greater “weight” in the synthesis because their findings were more trustworthy [24]. However, if poorer quality papers supported findings of higher quality papers their data were included.

### 3.3. Study Characteristics

One study [20] used qualitative methods, seven [16,17,18,19,21,22,23] used quantitative methods and one [25] used a mixed methods design. Three [17,19,20] were conducted in the UK, three [16,18,21] in the USA, one [25] in Brazil, one [23] in Canada, and one [22] in Switzerland (see Table 2). Studies were categorized by harm for analysis.

Papers about pain [20,23,25] used validated pain instruments or were validation studies for pain measures. Measures were designed for healthcare professionals to identify and rate pain. Interview data included patients’/parents’ and healthcare professionals’ perceptions of pain and its treatment.

**Table 2:** Characteristics of included studies.

Reference #	First Author & Date	Journal	Aim	Sampling Method	*n* =	Harm	Location	Data Collection Method	Analysis Method	Quality Rating
[17]	Anthony (2010)	Journal of Tissue Viability, 19, 98–105.	To compare three risk assessment scales with respect to predictive validity	Review	Pediatrics: 236	Skin	England	Comparing patient data	Mann Whitney and logistic regression	75%
[20]	Byrne (2001)	Journal of Psychometric Research, 50, 69–76.	(i) how pediatric nurses construed their patients’ pain; (ii) how these constructions were related to the emotional challenge of pain; and, (iii) how they influenced nurses’ communication with patients and specifically their management of pain.	Opportunistic	nurses: 13 Pediatrics: 16	Pain	England	Observations and interviews	Grounded theory	100%
[16]	Curley (2003)	Nursing Research, 52(1), 22–33.	(a) Establish the predictive validity of the Braden Q Scale in an acutely ill pediatric population; (b) determine the critical cut-off point for classifying patient risk; and (c) determine the best time to assess patient risk.	Convenience	Pediatrics: 90	Skin	USA	Comparing patient data	Parametric and nonparametric statistics were used	50%
[25]	Linhares (2012)	Brazilian Journal of Medical and Biological Research, 45, 1287–1294.	To examine the prevalence, assessment, and management of pediatric pain in a public teaching hospital.	Opportunistic	Infants: 70 Children: 36 Adolescents: 15	Pain	Brazil	Questionnaires and interview	Systematic categorical analysis and descriptive statistics	50%
[21]	Noonan (2011)	Journal of Pediatric Nursing 2006, 21(6), 445–453.	The purpose of this paper was to describe the spectrum of alterations in skin integrity and skin care needs of hospitalized infants and children.	Convenience	Pediatrics: 252	Skin	USA	Skin integrity audit tool and the Braden Q scale	Descriptive Statistics	100%
[22]	Schluer (2009)	Child and Adolescent Health, 18, 3244–3252.	The aim of the current study is to describe the frequency of pressure ulcers in a pediatric care setting and to identify the population at risk, as well as to assess the factors predisposing to the development of pressure ulcers.	Convenience	Children: 155	Skin	Switzerland	Direct systematic inspection of the skin, and a valid risk assessment instrument: Braden Scale	Descriptive and univariate statistical methods.	100%
[18]	Suddaby (2005)	Pediatric Nursing, 31(2), 132–138.	To develop a simple, single-page measurement tool that evaluates risk of skin breakdown in the pediatric population and apply it to the acutely hospitalized child	Not specified	Children: 347	Skin	USA	Risk assessment instrument: The Starkid Skin Scale	Descriptive statistics and unconditional logistic regression	75%
[23]	Taylor (2008)	Pain Research Management, *13*: 25–32	To highlight areas of good practice, identify areas for improvement, and inform development of hospital standards, education, future audits, and the research agenda.	Not specified	Children: 241	Pain	Canada	Interviews and pain assessments.	Statistical analysis was performed, including nonparametric tests.	100%
[19]	Willock (2009)	Journal of Wound Care, 18(1), 17–21	To develop a predictive pressure ulcer risk assessment scale based on patient data.	Prospective	Children: 265	Skin	England	Questionnaire, survey, and interview	Chi-square tests.	75%

Studies about skin integrity [16,17,18,19,21,22] included risk assessments of pressure ulcer development using scoring subscales. Some focused within the hospital setting and assessed the potential risk and characteristics of pressure ulcers; others involved validation or comparative analyses of different extant tools, and tested their specificity, sensitivity, and significance.

### 3.4. Synthesis

The results of included studies generated the following themes.

#### 3.4.1. Mistrust of Pain Reports

According to the patients and parents/caregivers (henceforth “parents”) whose accounts were sought in the included papers, pain was prevalent among patients but often went unrecorded by healthcare professionals [23,25]: “*Medical and nursing charts were analysed for 118 (97%) of the 12 patients. No notations were found regarding pain in the majority of charts*” (author) [25]. A number of reasons for this were raised. Nurses revealed mistrust of patient accounts: “*Although most attributions implied that complaints were unjustified, some went further in implying pretence. One child* ‘*deserved an Oscar for her performance*’” (author and nurse) [20].

If pain reports were perceived as unjustified by staff, the implication that they were fabricated or exaggerated by children was associated with a moralization of pain. Patients were judged as “*one of those kids*” (participant) [20]. If children refrained from reporting pain they were considered “good”: “*She has been very good, she hasn’t complained of any pain since coming off the morphine*” (nurse participant) [20].

Parents were not always judged as trustworthy observers either: “I don’t think he’s in a lot of pain, but his dad seems to think he is. But I mean you can tell if a child’s in pain. No, he’s fine.” (nurse participant) [20]. In contrast, physicians were more likely to assess pain based on the patient’s self-report or parental judgement [25].

This mistrust of patient/parent reports of pain suggested that nurses required a clinical assessment of pain for it to be considered valid.

#### 3.4.2. Validated Pain Measures and Treatment of Pain

Engagement with validated measures was poor: “*Facial activation … was less often reported by professionals (physicians = 7% of patients; nurses = 9% of patients)*” (author) [25]. A number of pain measures were available (e.g., Neonatal Facial Coding System, COMFORT Scale) but they tended to be used most by those who had received specialist training, e.g., Acute Pain Service (APS) teams [23]. Reluctance to use validated measures was mirrored by a reticence to give pain relief. When it was given it tended to be irregular: “*… in the preceding 24 h, 42% of the children who had experienced pain during admission had received no analgesia, 33% had received it intermittently and only 25% had received regular analgesia*” (author) [23]. Administering appropriate treatment was observed in APS teams, in departments using validated measures, and in surgical wards [23].

Among parents, a preference for pharmacological treatments was expressed [25]. This suggested that physiological change through drugs was perceived as more effective than non-pharmacological interventions. Furthermore, physicians were less likely to report non-pharmacological interventions, implying it was insignificant and there was no need for them to be recorded [25].

#### 3.4.3. Device-Related Injury

Equipment used in hospitals threatens skin integrity. Pressure ulcers can be caused by a number of medical devices and therapeutic aids including: intravenous catheter hubs, leg casts, electrodes, saturation probes, wheelchairs, and unadjusted prostheses [16,17,18,19,21,22]. Patients’ skin integrity was most at risk in hospital: “*Only a marginal number of pressure ulcers (n = 2%, 3%) developed at home, with most (n = 45%, 78%) developing on the ward where the child was hospitalized*” (author) [22].

#### 3.4.4. Vigilance and Communication

Prevention of pressure ulcers requires vigilance. Neonates and younger children are a high-risk group because their immature skin is more susceptible to pressure ulcers and because they are unable to communicate [22]. In neonatal care, immobility poses further risk [22] and in intensive care, patients of any age are unlikely to be able to communicate discomfort [18]. The studies reviewed suggested that ulcers were most commonly located on the ear and sacrum [17,22] although individual differences in skin integrity were observed. Pressure ulcers observed in included papers were ranked grade 1, the least severe according to the European Ulcer Advisory Panel [26].

#### 3.4.5. Tissue Viability Risk Assessment Tools

The most commonly used risk assessment tool report in included studies was the Braden Q [16,17,18,19,21,22], and when others were used they were compared to it, e.g., Glamorgan and Garvin scales [19]. Two papers [16,19] tested the specificity and sensitivity of the Braden Q and Glamorgan scales but comparative analysis of prediction accuracy was not possible due to the use of inconsistent score values. Additionally, inter-rater reliability of measures needs to be evaluated.

Further research with pediatric populations is required; assessments used in included papers were modified from adult data, except for one that used the Glamorgan scale with statistically modelled data from representative hospitalized pediatric patients [19]. Nevertheless, new data sets based purely on pediatric settings are needed.

### 3.5. Discussion

This review has identified a small evidence base supporting the use of pain, skin integrity, and extravasation as key harms for a CYPST. It has also identified a need for further research.

Pain was under-recorded and poorly treated because staff did not appear to trust patient/parent reports. This may be due to a misunderstanding or lack of communication when dealing with unsettled children exhibiting signs of pain. Parents who are struggling to settle their child believe the child is in pain; staff are then called who successfully use distraction techniques to help settle the child which indicates to them the child was not in pain. The truth may be somewhere between. However, if nurses explained their use of distraction techniques to parents it would help them understand and provide them with the skills to manage it differently in the future. Staff were also reticent about using validated measures. Education about such measures and increased availability in hospitals is required for their large-scale adoption. If staff are made aware that these tools measure the physical, psychological, and contextual aspects of pain perhaps their reticence to use them and mistrust of patient/parent reports would decline. In the UK, there is an emphasis on involving patients/parents within the multidisciplinary team [27,28]. For this to happen, a more family-centered approach is required; staff must listen to patients/parents and if reports of pain appear unjustified they must work together to identify the problem.

In the UK, the cost of treating pressure ulcers (in adults) is estimated between £1.4 billion each year [29]. Studies included in this review confirmed their prevalence among pediatric patients illustrating the need for greater vigilance and prevention [16,18,22]. Awareness of pressure ulcer risk must be increased for staff to engage in appropriate preventative care [30], e.g., repositioning the patient, adjusting prostheses or ortheses [31]. The best time to assess pressure ulcer risk is within 24 h of admission [16]. This suggests that staff may not have sufficient knowledge of the patient to complete the Braden Q risk assessment [16]. Rather, an easily accessible, fast, user-friendly tool is required. The CYPST may prove to be such a tool but further evidence of the ease and efficiency of identifying and monitoring these harms using the CYPST is required before such a judgment could be made. The majority of risk assessment tools have been developed in an ad hoc fashion rather than through rigorous data collection and analysis [19]. This review has confirmed that further pediatric-specific data are required to ensure any new or existing tools are sufficiently sensitive and appropriate for pediatric settings.

A systematic review [15] was identified in the search which examined the pediatric alert criteria (PAC) used in PEWS tools. The PEWS were designed to identify potential deterioration and to trigger appropriate corrective action. However, none of the studies included in that review evaluated the ease or efficiency of use in a busy pediatric setting, nor did they assess staff satisfaction with them. Evidence of their clinical utility varied and only one [10] was judged to meet the recommendations of the UK National Institute for Health and Care Excellence (NICE) [15], the standards by which services are endorsed for provision in the NHS. The review concluded that although evidence in favor of PEWS was identified further research was required to fully examine their use. An evaluation of an Early Warning System (EWS) in adult care [32] could be used as a model of what is required in pediatric settings. This was an in-depth qualitative study of nurses’ experiences of using the EWS which found that it improved communication between healthcare professionals, it gave nurses a transparent and common language with which to communicate concern, and empowered them to manage potential deterioration quickly and appropriately.

The ST has shown improved safety in adult settings with increasing “harmfree care” scores on a monthly basis [33]. Furthermore, staff have been reported to appreciate the benefit of the ST because it facilitates a more in-depth analysis of harms and offers printed graphical data [32,34]. The positive response to the ST shows promise for a pediatric specific version because the change process has already been implemented within adult settings [32,33,34,35]. Nevertheless, the results of this review demonstrate a shift in perceptions among staff is required for them to engage in using standardized measures or tools. Furthermore, the importance of a family-centered approach has been emphasized by the challenges evoked through an inability to communicate directly with neonates and younger children. Staff need to work together with parents to ensure an appropriate judgment regarding deterioration and the escalation of care is made and that care is both suitable and effective.

### 3.6. Limitations

This review identified a relatively small number of studies demonstrating a lack of evidence in the field. Some studies were poorer quality due to a lack of transparency in methods used and sometimes the rationale for those methods. Transparency of reporting is central to establishing the trustworthiness of findings [36]; better reporting in studies conducted in healthcare services would improve the quality of the evidence base. Although risk assessment and PEWS tools have been developed and tested to a degree, research is still at the developmental phase which means there was a lack of intervention studies to assess the effectiveness of their use in practice. There was also a distinct lack of emphasis on assessing these harms as triggers to escalate care and when those triggers fail.

The results of this mixed studies review are therefore to be considered cautiously because of the limitations of the included studies and relative infancy of research in this field.

## 4. Conclusions

The ST has shown initial improvement in safety audits in adult care. Thus, the argument for a similar tool in pediatric settings is supported. This review has identified evidence in favor of the use of pain and skin integrity in a pediatric safety tool; extravasation could be considered as a subgroup of skin integrity but because of the high recorded incidence of harm associated with this device and the differences in the clinical management of extravasations, pressure ulcers, and moisture lesions, clinical experts propose that it should be kept separate. The use of PEWS tools was evaluated elsewhere [15] but the conclusion was that, like the other harms, further evidence of their utility and staff satisfaction of using them was required. The literature proposes that the next steps should involve further data generation specific to pediatric settings to enable further validation work with existing and new risk assessment and PEWS tools. In addition, further research is required to examine safety of drug administration within pediatric settings alongside work with healthcare professionals and families to assess the acceptability and feasibility of implementing the CYPST. Key issues for that research would be the observed mistrust and poor communication between staff and patients/parents, staff’s reticence to use standardized tools, and the potential for the ST to provide clarity and improved communication and thus more accurate, quicker care decisions. Furthermore, there is now a need to focus on establishing the outcome measures of the CYPST in order to determine its effectiveness in the future.
